# Psychopathy and Sadism: A Systematic Review of Their Association, Core Dimensions, and Nosological Implications

**DOI:** 10.7759/cureus.112155

**Published:** 2026-07-06

**Authors:** Néstor Quinapanta-Castro, Andrea G Suárez López, Fredy Alonso Loaiza Valencia, Félix Guerrero Quiñonez, Lizeth Morales, Mayra López, Erick Alejandro Tamayo-Vanegas

**Affiliations:** 1 Department of Doctoral Studies, Faculty of Philosophy and Letters, Universidad de Buenos Aires, Buenos Aires, ARG; 2 Department of Psychology, Faculty of Medical Sciences, Universidad Regional Autónoma de Los Andes, Ambato, ECU; 3 Department of Doctoral Studies, Faculty of Jurisprudence and Social Sciences, Universidad de Salamanca, Salamanca, ESP; 4 Department of Obstetrics and Gynecology, Faculty of Medical Sciences, Universidad Regional Autónoma de Los Andes, Ambato, ECU; 5 Department of Obstetrics and Gynecology, Hospital Básico de Pelileo, Pelileo, ECU; 6 Department of Medicine, Faculty of Medical Sciences, Universidad Regional Autónoma de Los Andes, Ambato, ECU; 7 Department of Medicine, Faculty of Medical Sciences, Universidad Central del Ecuador, Quito, ECU

**Keywords:** adults aged 18+, antisocial personality disorder, psychopathy, sexual sadism, sexual sadism disorder

## Abstract

This systematic review aimed to synthesize the available scientific evidence on the relationship between psychopathy and sadism. It was conducted in accordance with the PRISMA 2020 guidelines and prospectively registered in the International Prospective Register of Systematic Reviews (PROSPERO, CRD420261394636). Included studies had to assess both constructs using validated instruments such as the Psychopathy Checklist-Revised (PCL-R), the Self-Report Psychopathy Scale (SRP), the Levenson Self-Report Psychopathy Scale (LSRP), the Psychopathic Personality Inventory-Revised (PPI-R), or the Triarchic Psychopathy Measure (TriPM) for psychopathy and clinical or behavioral scales for sadism or sexual sadism. A literature search was performed in PubMed/MEDLINE, Scopus, and Web of Science using terms related to psychopathy, sexual violence, sexual aggression, and sadism, combined with Boolean operators. Two reviewers independently performed study selection and data extraction, while methodological quality was assessed using the Newcastle-Ottawa Scale (NOS), Cochrane Risk of Bias version 2 (RoB 2), and AMSTAR-2 (A Measurement Tool to Assess Systematic Reviews 2) tools. The certainty of the evidence was evaluated using the GRADE framework.

According to the PRISMA flowchart, 1,061 records were identified. After removing 239 duplicates and applying the selection criteria, 18 studies (19 reports) involving 12,302 participants with a mean age of 34.01 ± 9.98 years were included. The evidence consisted of 16 observational studies and two meta-analyses. The studies primarily focused on forensic, clinical, and prison populations, and there was a predominant representation from Europe and the Americas (42.11% each). The studies used validated instruments, such as the PCL-R, LSRP, SRP, and TriPM, as well as specific scales for sexual and everyday sadism.

Meta-analyses identified moderate to significant positive correlations between global psychopathy and sadism (r = 0.24, 95% CI: 0.16-0.34, p < 0.001) and between psychopathy and sexual sadism (r = 0.33, 95% CI: 0.21-0.44, p < 0.001). However, there was high heterogeneity (I² = 84.5-92.3%). Individual studies revealed positive correlations between psychopathy and sadism ranging from low to moderate (r = 0.16-0.53). However, some studies found no significant associations.

Individuals with sadistic characteristics generally had higher psychopathy scores than non-sadistic subjects. Significant differences in PCL-R scores were observed between sadistic and non-sadistic groups (21.49 vs. 17.12; p = 0.004; d = 0.59) and between sexual offenders with and without sadism (27.0 vs. 19.0; p = 0.01). Nevertheless, some findings were contradictory, with studies failing to identify a specific relationship between psychopathy and sexual sadism.

Analysis of the psychopathic dimensions revealed that the antisocial, impulsive, and affective components exhibited the strongest associations with sadism. The antisocial facet predicted sexual aggression (r = 0.48, β = 0.26, p < 0.001), and the affective dimension showed significant associations with sexual sadism (β = 0.27). In dimensional models, "malice" and disinhibition were the components most strongly related to sadism, while audacity showed weak associations. Among a non-forensic population, latent correlations between psychopathy and everyday sadism were very high (ρ = 0.94-0.98), suggesting a possible conceptual overlap between the two constructs.

## Introduction and background

Psychopathy is a personality disorder characterized by a cluster of affective, interpersonal, lifestyle, and antisocial traits. The precursors of these traits can be identified in a subgroup of young people exhibiting severe antisocial behavior [1]. Recent meta-analytic evidence estimates the prevalence of psychopathy in the general adult population at 4.5%; however, this estimate varies by assessment method. When evaluated with the Psychopathy Checklist-Revised (PCL-R), the prevalence decreases to approximately 1.2%, suggesting that severe clinical psychopathy is substantially less common than broader self-reported psychopathic traits [2]. It is a significant risk factor strongly associated with homicides, particularly severe forms such as sexual, sadistic, serial, and multiple-perpetrator homicides [3]. Furthermore, individuals with psychopathic traits tend to die at younger ages than the general population, and their deaths are often more violent [4]. Numerous studies have examined the relationship between psychopathy and sexual offending, with a focus on violence, aggression, and coercion as antisocial risk factors [5,6].

On the other hand, sadism is defined as the tendency to derive pleasure from the suffering of others [7]. Within this construct, sexual sadism is defined as a sexual preference disorder characterized by obtaining sexual arousal or gratification through inflicting suffering, pain, or humiliation on another person [8]. It is not limited to violent criminals but can also occur in nonclinical and noncriminal populations [9]. Sexual sadism is primarily identified among sexual offenders, especially sexual homicide offenders. Prevalence rates are as high as 75% among this group, compared to 4-42% among general sex offenders and 2.3-12% among non-forensic clinical samples [10]. Individuals with sadistic traits derive pleasure not only from observing others’ pain, but also from actively inflicting it. They are motivated by both the enjoyment of the aggressive act and the suffering caused [11].

Both constructs share conceptual space with the Dark Tetrad of personality traits (narcissism, Machiavellianism, psychopathy, and sadism) [9,12], but their specific relationship remains the subject of empirical debate. Some authors argue that sadism is a functionally independent component that increases the violent potential of psychopathy [13,14], while others claim that they are overlapping phenotypes with shared neurobiological bases, such as deficits in processing fear or others’ pain, as well as deficits in amygdala activation in response to distress signals [15-18].

This study's theoretical rationale rests on the relevance of both constructs to explanatory models of serious violence. Psychopathy is one of the strongest predictors of violent recidivism [19,20], while sadism has been identified as a distinguishing factor between sexual offenders with and without lethal victims [21,22]. Therefore, understanding how these two dimensions interact is essential for developing more accurate risk assessment tools and designing evidence-based prison intervention programs.

Finally, this review addresses the ongoing debate about whether sadism should be considered a diagnostic indicator within antisocial or psychopathic personality disorders or an independent dimension in contemporary dimensional taxonomies, such as the HiTOP (Hierarchical Taxonomy of Psychopathology) model or the Diagnostic and Statistical Manual of Mental Disorders, 5th Edition (DSM-5) Section III [23] framework for personality disorders. Resolving this issue has implications for psychiatric nosology and translational research in forensic neuroscience.

Given the heterogeneity of populations, assessment methods, and theoretical frameworks in the existing literature, as well as the absence of a comprehensive synthesis integrating both clinical and subclinical manifestations of psychopathy and sadism, this systematic review aimed to critically evaluate the available evidence on the association between these constructs, identify the psychopathic dimensions most consistently linked to sadistic tendencies, and examine whether current empirical findings support their conceptual distinctiveness or indicate substantial overlap.

## Review

Methodology

This study was conducted as a systematic review in accordance with the Preferred Reporting Items for Systematic Reviews and Meta-Analyses (PRISMA) 2020 guidelines [24]. The protocol was prospectively registered with the International Prospective Register of Systematic Reviews (PROSPERO) under the registration number CRD420261394636. The review aimed to synthesize the available evidence regarding the association between psychopathy and sexual sadism in adult populations in forensic, psychiatric, correctional, and community settings.

Research Question

The review was guided by the following primary research question: What is the association between sadism or sexual sadism and psychopathy in adult populations according to the available scientific literature? Two secondary questions were also explored: Which psychopathic dimensions or traits are most strongly associated with sexual sadism? How has the relationship between psychopathy and sadism been operationalized and measured across studies?

Eligibility Criteria

Observational and experimental studies, as well as meta-analyses, evaluating the association between psychopathy and sadism in adult forensic or community populations were included. Eligible studies were required to assess both constructs using validated instruments and to report quantitative data allowing estimation of their association. Studies conducted exclusively in adolescents, those that did not simultaneously assess psychopathy and sadism or failed to report relevant outcomes, as well as narrative reviews, editorials, letters, protocols, case reports, duplicate publications, and articles without accessible full text, were excluded. Studies that failed to assess both psychopathy and sadism using validated methods were also excluded.

Psychopathy was assessed using validated psychometric or clinical instruments, such as the Psychopathy Checklist-Revised (PCL-R), the Self-Report Psychopathy Scale (SRP), the Levenson Self-Report Psychopathy Scale (LSRP), the Psychopathic Personality Inventory-Revised (PPI-R), the Triarchic Psychopathy Measure (TriPM), or standardized Diagnostic and Statistical Manual of Mental Disorders (DSM)/International Classification of Diseases (ICD) diagnostic criteria. Sadism measures included clinical diagnoses, validated self-report instruments, structured interviews, and behavioral or experimental paradigms. Instruments used across studies included the Short Sadistic Impulse Scale (SSIS), the Assessment of Sadistic Personality (ASP), the Comprehensive Assessment of Sadistic Tendencies (CAST), Varieties of Sadistic Tendencies (VAST), the Severe Sexual Sadism Scale (SSSS), the Sexual Sadism Assessment Scale (SESAS/SeSaS), the Multidimensional Inventory of Development, Sex, and Aggression (MIDSA) Sadistic Fantasies and Behaviors Scales, as well as measures of sexual aggression and coercion, sadistic fantasies, specialized forensic classification systems (Massachusetts Treatment Center: Revised Rapist Typology, Version 3 (MTC:R3)/Massachusetts Treatment Center: Child Molester Typology, Version 3 (MTC:CM3)), Diagnostic and Statistical Manual of Mental Disorders, 4th Edition (DSM-IV) or Diagnostic and Statistical Manual of Mental Disorders, 4th Edition, Text Revision (DSM-IV-TR)-based diagnostic evaluations, and experimental paradigms designed to assess sadistic and aggressive behaviors.

Information Sources and Search Strategy

In accordance with the PRISMA 2020 guidelines [24], a comprehensive literature search was conducted using the electronic databases PubMed/MEDLINE, Scopus, and Web of Science. The search strategy combined controlled vocabulary terms, such as MeSH and DeCS (Descriptores en Ciencias de la Salud), and free-text keywords related to psychopathy, psychopathic traits, sexual sadism, sexual violence, and sexually aggressive behavior. The search terms were adapted to the indexing systems and syntax requirements of each database.

To maximize sensitivity and capture the broadest possible range of relevant studies, the search strategy incorporated terms associated with sexual offending, sexually coercive behavior, and paraphilic interests. These included: “sex offender,” “sexual violence,” “sexual homicide,” “sexual aggression,” “sexual coercion,” “sexual victimization,” “sexual perpetration,” “sexual deviance,” “sexual abuse,” “rape,” “forced sex,” “sexual assault,” “pedophilia,” “child sexual abuse,” “paraphilia,” “sexual sadism,” “exhibitionism,” “indecent exposure,” “sex trafficking,” “internet sex crimes,” and “intimate partner sexual violence.” Truncation symbols and wildcard operators were applied where appropriate to increase retrieval sensitivity.

These terms were combined using Boolean operators (AND, OR) with psychopathy-related search terms, including “psychopathy,” “psychopathic traits,” “Psychopathy Checklist,” “Psychopathy Checklist-Revised (PCL-R),” “Triarchic Psychopathy Measure (TriPM),” “Levenson Self-Report Psychopathy Scale (LSRP),” “Self-Report Psychopathy Scale (SRP),” “Psychopathic Personality Inventory (PPI-R),” “Interpersonal Measure of Psychopathy,” “Elemental Psychopathy Assessment,” and personality-based psychopathy measures derived from the HEXACO and NEO personality frameworks.

The search strategy was intentionally broad to capture clinical and subclinical forms of sexual sadism, as well as studies involving forensic, psychiatric, correctional, university, and community populations. To identify additional studies, the reference lists of the included articles were manually screened. Contacting authors or subject-matter experts was also considered a supplementary method for identifying additional relevant studies.

Study Selection

All the references retrieved were exported to reference management software, where duplicate records were identified and removed. The screening process was conducted in two phases by two reviewers working independently. During the first phase, the titles and abstracts of the references were evaluated according to the predefined eligibility criteria. In the second phase, the full texts of the articles were assessed to determine final inclusion.

Any disagreements between the reviewers were resolved through discussion and consensus. The entire selection process was documented using a PRISMA flow diagram to ensure transparency and reproducibility.

Data Extraction

Two reviewers independently performed data extraction using a standardized form. The extracted data included authorship, publication year, study design, sample characteristics, assessment instruments, and main findings concerning the relationship between psychopathy and sadism.

Outcomes

The primary outcomes were aligned with the primary association between psychopathy and sadism examined in each study. The secondary outcomes included additional analyses, such as contributions of specific psychopathic traits, comparisons between subgroups, predictive models, and correlation analyses.

Risk of Bias and Quality Assessment

The methodological quality and risk of bias were evaluated based on the study design. Observational studies were evaluated using the Newcastle-Ottawa Scale (NOS) [25], which assesses study selection, comparability, and exposure or outcome assessment. Scores ranging from 7 to 9 were considered high quality; scores between 5 and 6, moderate quality; and scores of 4 or lower, low quality.

Experimental studies were evaluated using the Cochrane Risk of Bias version 2 (RoB 2) [26], which examines randomization, deviations from intended interventions, missing outcome data, outcome measurement, and selective reporting.

The included meta-analyses were evaluated using the AMSTAR-2 (A Measurement Tool to Assess Systematic Reviews 2) tool [27]. This instrument evaluates the methodological rigor of systematic reviews, including the registration of protocols, the execution of systematic searches, the selection of studies and extraction of data in duplicate, the assessment of risk of bias, the application of statistical methods, the analysis of publication bias, and the disclosure of conflicts of interest.

Certainty of Evidence

The certainty of the evidence was assessed using the Grading of Recommendations, Assessment, Development, and Evaluation (GRADE) approach [28]. This framework classifies the overall quality of the evidence into one of four levels - high, moderate, low, or very low - based on five domains: the risk of bias of the included studies; the consistency of the findings across the studies; the directness of the evidence in relation to the research question; the precision of the results; and the potential for publication bias.

Data Synthesis

A narrative and thematic synthesis was conducted due to the expected methodological heterogeneity among the studies.

Results

**Figure 1:**
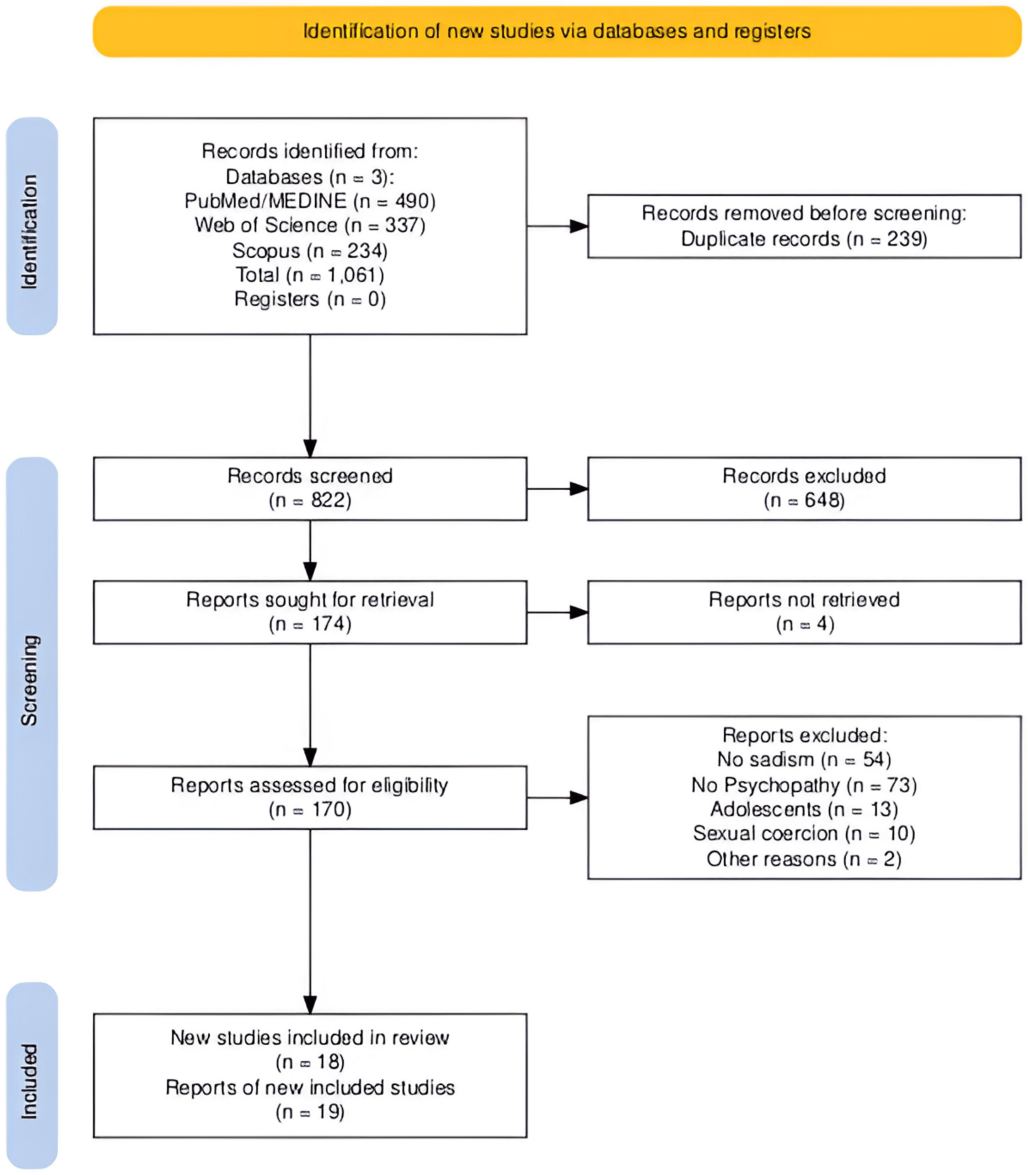
PRISMA flow diagram. PRISMA: Preferred Reporting Items for Systematic Reviews and Meta-Analyses.

In accordance with the PRISMA guidelines, a total of 1,061 records were identified across three databases: PubMed/MEDLINE, Web of Science, and Scopus. After 239 duplicates were removed, 822 studies were screened, of which 648 were excluded. Of the 174 articles reviewed, four could not be obtained. Then, a total of 170 full-text articles were evaluated, primarily excluding studies lacking psychopathy or sadism data and studies including adolescents. Eighteen studies (19 reports) were ultimately included in the systematic review (Figure 1).

A total of 12,302 participants were represented across the included studies. The mean age of the participants reported in studies providing age data was 34.01 ± 9.98 years (Table 1). Participants were drawn from 16 primary studies [13,18,29-42] and two meta-analyses [14,43]. This makes the study one of the most comprehensive bodies of research on the relationship between psychopathy and sexual aggression to date.

**Table 1:** Characteristics of Studies Examining the Relationship Between Psychopathy and Sadism PCL-R: Psychopathy Checklist-Revised; SRP: Self-Report Psychopathy Scale; LSRP: Levenson Self-Report Psychopathy Scale; PPI-R: Psychopathic Personality Inventory-Revised; TriPM: Triarchic Psychopathy Measure; SD4: Short Dark Tetrad; IPDE: International Personality Disorder Examination; SSIS: Short Sadistic Impulse Scale; ASP: Assessment of Sadistic Personality; CAST: Comprehensive Assessment of Sadistic Tendencies; VAST: Varieties of Sadistic Tendencies; SSSS: Severe Sexual Sadism Scale; SeSaS: Sexual Sadism Scale; SESAS: Severe Sexual Sadism Scale; RCAP: Response Choice Aggression Paradigm; SAG: Severity of Aggression rating; MASA: Multidimensional Assessment of Sex and Aggression; MIDSA: Multidimensional Inventory of Development, Sex, and Aggression; MTC:R3: Massachusetts Treatment Center: Revised Rapist Typology, Version 3; MTC:CM3: Massachusetts Treatment Center: Child Molester Typology, Version 3; MCMI-II: Millon Clinical Multiaxial Inventory-II; PDE: Personality Disorder Examination; SPD: Sadistic Personality Disorder; DSM-IV-TR: Diagnostic and Statistical Manual of Mental Disorders, 4th Edition, Text Revision; VPCO: Violence Profile for Current Offense; BIS-11: Barratt Impulsiveness Scale-11; PRISMA: Preferred Reporting Items for Systematic Reviews and Meta-Analyses; SES: Sexual Experiences Survey; MASORR: Multifactorial Assessment of Sex Offender Risk for Recidivism; MSI: Multiphasic Sexual Inventory; SVR-20: Sexual Violence Risk-20; NEIS: Negative Emotional Intensity Scale; EEG: Electroencephalography.

Author/Year	Country	Mean ± SD	Design	Sample	Psychopathy instruments	Sadism/aggression instruments
Rosenberg et al. (2005) [29]	United States	36.09 ± 16.03	Retrospective cross-sectional	N = 111 child sexual offenders	PCL-R	Coding of physical violence/coercion
Reidy et al. (2011) [41]	United States	19.20 ± 1.50	Experimental laboratory	N = 137 male university students	SRP-III; LSRP	RCAP; experimental measures of sadism
Cardona et al. (2018) [30]	United States	40.21 ± 10.66	Cross-sectional correlational	N = 302 sexual offenders	PCL-R facets	SAG; MASA
Lassche et al. (2024) [40]	Netherlands	20.98 ± 2.54	Correlational with EEG	N = 50 young adults	SD4	Everyday sadism subscale
Woodworth et al. (2013) [31]	Canada	43.88 ± 11.24	Retrospective descriptive	N = 139 sexual offenders	PCL-R	Sexual fantasies; DSM-IV-TR diagnoses
Berger et al. (1999) [32]	Austria	35.50 ± 10.70	Cross-sectional correlational	N = 70 inmates	IPDE	SPD diagnosis and sexual sadism
Robertson et al. (2014) [33] - Study 1	United States	39.06 ± 9.66	Cross-sectional	N = 314 male inmates	PCL-R (4 facets)	MIDSA (self-report): Sadistic Fantasies Scale + Sadistic Behaviors Scale
Robertson et al. (2014) [33] - Study 2	United States	36.26 ± 11.54	Cross-sectional	N = 599 male inmates	PCL-R (4 facets)	MTC:R3/MTC:CM3 (archival classification)
Lobbestael et al. (2020) [42]	Netherlands	32.22 ± 16.53	Between-groups experimental	N = 120 community men	PPI-R	SSIS; bug-grinder paradigm
Darjee (2019) [34]	Scotland	26.30	Clinical cross-sectional	N = 51 sexual homicide offenders	PCL-R	DSM-IV; SeSaS
Holt et al. (1999) [35]	United States	30.73 ± 7.30	Factorial correlational	N = 41 inmates	PCL-R	MCMI-II 6B; PDE
Blötner et al. (2025) [18]	Germany	30.80 ± 10.80	Cross-sectional correlational	N = 1,076 students	LSRP; SRP-4; TriPM	VAST; CAST; ASP; SSIS
Packer et al. (2025) [43]	NA	32.14 ± 8.23	PRISMA meta-analysis	15 studies; N = 3,074	PCL-R; TriPM; PPI-R; LSRP	SES; MASORR; MSI; SVR-20
Mokros et al. (2010) [13]	Germany	36.00 ± 10.00	Observational correlational	N = 100 forensic patients	PCL-R four facets	SSSS
O’Connell et al. (2019) [14]	NA	—	Meta-analysis	19 samples; N = 5,161	PCL-R total and factors	Clinical and behavioral sadism scales
Papagathonikou et al. (2025) [36]	United Kingdom	41.01 ± 10.30	Cross-sectional correlational	N = 59 psychiatric patients; N = 6 participants incarcerated	PCL-R	SESAS; ASP
Balcioglu et al. (2023) [37]	Turkey	37.08 ± 11.89	Cross-sectional correlational	N = 64 sexual offenders	LSRP	VPCO index; BIS-11
Eher et al. (2015) [38] - Study 2	Austria	41.28 ± 12.69	Retrospective cohort	N = 768 sexual offenders	PCL-R	DSM-IV-TR; SeSaS
Brown et al. (1997) [39]	Canada	33.40 ± 8.13	Retrospective cross-sectional	N = 60 incarcerated rapists	PCL-R	MTC:R3; NEIS

Most of the included studies [13,29-39] were conducted in clinical, forensic, or correctional settings or through reviews of judicial and psychiatric records. These studies used samples of sex offenders, forensic psychiatric patients, or incarcerated individuals and employed clinical records, structured interviews, and risk assessment tools. In contrast, some studies [18,40-43] used nonforensic community or university samples to assess subclinical traits of psychopathy and sadism using self-reporting and experimental methods. Finally, O’Connell et al. [14] conducted a meta-analysis of previous studies on psychopathy and sexual sadism, incorporating both forensic and non-forensic samples, as shown in Table 1.

According to the 19 included records, the geographic distribution by continent shows that Europe and the Americas were the most represented regions, with eight cases in Europe (42.11%) and eight cases in the Americas (42.11%), mainly from the United States and Canada. With a single case (5.26%), Asia is the least represented region, with Turkey being the only country included in this category. Finally, two records (10.53%) correspond to meta-analyses without a defined continental location.

Association Between Psychopathy and Sadism

This systematic review consistently shows a positive and statistically significant association between psychopathy and sadism in both forensic samples and subclinical populations, as shown in Table 2. However, the magnitude of the effect varies depending on the design of each study.

**Table 2:** Associations between psychopathy and sadism across studies. PCL-R: Psychopathy Checklist-Revised; PCL: Psychopathy Checklist; SRP: Self-Report Psychopathy Scale; SRP-III: Self-Report Psychopathy Scale, Version III; SRP-4: Self-Report Psychopathy Scale, Version 4; LSRP: Levenson Self-Report Psychopathy Scale; PPI: Psychopathic Personality Inventory; PPI-R: Psychopathic Personality Inventory-Revised; SCI: Self-Centered Impulsivity (PPI-R subscale); FD: Fearless Dominance (PPI-R subscale); TriPM: Triarchic Psychopathy Measure; SD4: Short Dark Tetrad; SSIS: Short Sadistic Impulse Scale; ASP: Assessment of Sadistic Personality; AST: Assessment of Sadistic Traits; CAST: Comprehensive Assessment of Sadistic Tendencies; VAST: Varieties of Sadistic Tendencies; SSSS: Severe Sexual Sadism Scale; SeSaS: Sexual Sadism Scale; DSM: Diagnostic and Statistical Manual of Mental Disorders; DSM-III-R: Diagnostic and Statistical Manual of Mental Disorders, Third Edition, Revised; DSM-IV: Diagnostic and Statistical Manual of Mental Disorders, Fourth Edition; DSM-IV-TR: Diagnostic and Statistical Manual of Mental Disorders, Fourth Edition, Text Revision; ICD-10: International Classification of Diseases, 10th Revision; SPD: Sadistic Personality Disorder; ASPD: Antisocial Personality Disorder; MIDSA: Multidimensional Inventory of Development, Sex, and Aggression; MCMI-II: Millon Clinical Multiaxial Inventory-II; PDE: Personality Disorder Examination; MTC:R3: Massachusetts Treatment Center: Revised Rapist Typology, Version 3; SVR-20: Sexual Violence Risk-20; VRAG-R: Violence Risk Appraisal Guide-Revised; VRS-SO: Violence Risk Scale-Sexual Offender version; SORAG: Sex Offender Risk Appraisal Guide; MnSOST-R: Minnesota Sex Offender Screening Tool-Revised; RM2000/S: Risk Matrix 2000/Sexual; VPCO: violence during perpetration of the sexual offense index; VIF: variance inflation factor; CI: confidence interval; AUC: area under the curve; OR: odds ratio; EEG: electroencephalography; NEIS: Negative Emotional Intensity Scale.

Author	Primary results	Secondary results
Rosenberg et al. (2005) [29]	Child sexual offenders who used physical violence during the sexual offense had significantly higher PCL-R scores than those who did not (M = 22.79 vs. 15.55; p < 0.0001).	Both PCL-R factors (F1 affective-interpersonal and F2 antisocial) were significantly associated with violence during the sexual offense (p < 0.0001).
Reidy et al. (2011) [41]	Psychopathy (LSRP and SRP-III) and sadism (Lexical Decision Task): The overall model was not statistically significant (F = 1.95; R² = 0.03; p > 0.10), indicating that psychopathic traits accounted for only 3% of the variance in sadism.	Psychopathy (LSRP and SRP-III) and sadism (Lexical Decision Task): F1 was not associated with sadism (b = 0.11; p = 0.10). F2 was negatively associated with sadism (b = −0.21; p = 0.05).
Cardona et al. (2018) [30]		PCL-R scores: The antisocial facet (Facet 4) was the strongest predictor of aggression, being associated with both non-sexual aggression (r = 0.48, p < 0.001; β = 0.31, p < 0.001) and sexual aggression (r = 0.48, p < 0.001; β = 0.26, p < 0.001). The impulsive and lifestyle facet (Facet 3) was also related to non-sexual aggression (r = 0.40, p < 0.001; β = .21, p = 0.002), whereas the affective facet (Facet 2) was associated with sexual aggression (r = 0.28, p < 0.001; β = 0.18, p = 0.002). The interpersonal facet (Facet 1) did not show significant associations in the final models (p > 0.10).
Lassche et al. (2024) [40]	SD4: A moderate and significant positive correlation was found between psychopathy and everyday sadism (r = 0.53; p < 0.01).	
Woodworth et al. (2013) [31]	DSM-IV-TR and PCL-R: Psychopathy showed a specific association with sexual sadism (χ² = 14.89; p = 0.001). Individuals with sadism were more frequently characterized by high levels of psychopathy compared to those with moderate (χ² = 4.84; p = 0.03) and low levels (χ² = 21.80; p < 0.001), with no significant differences between low and moderate levels (p = 0.10). In descriptive terms, 67% of sadistic individuals exhibited high psychopathy, and 58% of individuals with high psychopathy presented sexual sadism.	
Berger et al. (1999) [32]	42.10% of patients with Sadistic Personality Disorder (SPD) met criteria for Antisocial Personality Disorder (ASPD), according to the DSM-III-R; however, this association did not reach statistical significance. In contrast, when ICD-10 criteria (dissocial disorder) were applied, the proportion increased to 57.90%, and a statistically significant association was observed in this case (p < 0.05; Fisher’s exact test).	Taken together, the factor structure did not support SPD as a discrete and independent diagnostic entity separate from antisocial personality disorder; rather, it positions it as a subdimension of the same construct, characterized by pervasive cruelty and a dominance-seeking orientation.
Robertson et al. (2014) [33]	PCL-R and MIDSA: In Study 1, sexual sadism correlated with total psychopathy (r = 0.35; p < 0.001). PCL-R and MTC:R3: In Study 2, a significant correlation was also observed (r = 0.16; p < 0.01).	Study 1 (PCL-R and MIDSA): associations with Interpersonal Facet 1 (r = 0.26; p < 0.001), Impulsivity Facet 3 (r = 0.28; p < 0.001), and Antisocial Facet 4 (r = 0.27; p < 0.001), but not with Affective Facet 2. Study 2 (PCL-R and MTC:R3): associations with Facets 1 (r = 0.14; p < 0.01), 2 (r = 0.10; p < 0.05), and 4 (r = 0.15; p < 0.01), but not with Facet 3 (r = 0.04; p > 0.05).
Lobbestael et al. (2020) [42]	PPI-R and SSIS: Dispositional sadism showed a significant positive correlation with total psychopathy (r = 0.25; p = 0.005).	Sadism was primarily associated with (SCI) (r = 0.40; p < 0.001), but not with Fearless Dominance (r = 0.04; p = 0.66) or Coldheartedness (r = −0.03; p = 0.74). In the regression analysis, SCI was the only significant predictor of self-reported sadism (β = 0.40; p < 0.001). In terms of behavioral sadism, coldheartedness was found to be the most significant factor, being linked to a greater number of crushed insects (r = 0.22; p = 0.02) and significantly predicting this behavior (OR = 1.08; Wald χ² = 6.60; p = 0.01). Additionally, it was associated with greater positive affect following aggression (β = 0.17; t = 2.40; p = 0.02), lower negative affect (β = −0.28; t = −3.23; p = 0.002), and diminished feelings of guilt (β = −0.24; t = −2.65; p = 0.009). In contrast, fearless dominance (FD) only predicted lower guilt (β = −0.30; t = −3.30; p = 0.001). SCI showed no significant associations with aggressive behavior, positive affect, negative affect, or guilt.
Darjee (2019) [34]	Offenders with DSM-IV sexual sadism scored higher on the PCL-R than non-sadistic offenders (27.0 vs. 19.0; θ = 0.72; p = 0.01). SeSaS correlated significantly with total PCL-R (τ = 0.22; p = 0.04).	Factor 1 of the PCL-R exhibited a notable correlation with the dimensional measure of sexual sadism (τ = 0.24; p = 0.03), whereas its correlation with the DSM-IV diagnosis was weaker and only marginally significant (θ = 0.64; p = 0.06). By contrast, Factor 2 exhibited the strongest correlation with the categorical DSM-IV diagnosis of sexual sadism (θ = 0.70; p = 0.02), though its correlation with the SeSaS did not reach statistical significance (τ = 0.17; p = 0.13).
Holt et al. (1999) [35]	Psychopaths scored (PCL-R) significantly higher on trait sadism than non-psychopaths (83.60 vs. 58.50; p = 0.004) on the MCMI-II Scale 6B and on the PDE (8.45 vs. 2.84; p = 0.0001).	MCMI-II Scale 6B correlated significantly with PCL-R Factor 1 (r = 0.45; r² = 0.20; p < 0.008) and Factor 2 (r = 0.46; r² = 0.21; p < 0.008), but not with DSM-IV sexual sadism (r = 0.17; r² = 0.03). PDE correlated significantly with Factor 1 (r = 0.64; r² = 0.41; p < 0.008) and Factor 2 (r = 0.57; r² = 0.32; p < 0.008).
Blötner et al. (2025) [18]	Latent correlations between subclinical psychopathy (LSRP, SRP-4, and TriPM) and everyday sadism (AST, CAST, ASP, and SSIS) were extremely high (ρ = 0.94 - 0.98; p < 0.001), suggesting possible conceptual overlap between the constructs. The SRP-4 scale correlated with ASP at r = 0.70, p < 0.001; with CAST at r = 0.68, p < 0.001; and with VAST at r = 0.56, p < 0.001. The LSRP showed correlations of r = 0.66 with ASP, p < 0.001; r = 0.59 with CAST, p < 0.001; and r = 0.47 with VAST, p < 0.001. Likewise, the TriPM correlated r = 0.65 with ASP, p < 0.001; r = 0.64 with CAST, p < 0.001; and r = 0.55 with VAST, p < 0.001.	In the TriPM model, “Meanness” was the dimension most strongly associated with sadism (ASP, r = 0.74; CAST, r = 0.62; VAST, r = 0.61; p < 0.001), followed by “Disinhibition” (CAST, r = 0.61; ASP, r = 0.55; VAST, r = 0.37), whereas “Boldness” showed weak associations (r = 0.10 - 0.18). Proactive aggression (r = 0.46 - 0.60) and reactive aggression (r = 0.30 - 0.47) were significantly associated with psychopathy and sadism (p < 0.001). Affective empathy showed strongly negative correlations (r = −0.64 - −0.76), whereas interpersonal dominance (r = 0.63 - 0.70) and Machiavellianism (r = 0.56 - 0.68) showed high and consistent associations, indicating a common core based on interpersonal cruelty, dominance, and empathic deficits.
Packer et al. (2025) [43]	The meta-analysis showed a moderate positive association between sexual sadism (DSM/clinical diagnostic criteria, forensic and prison records, and structured risk assessment instruments such as SVR-20, VRAG-R, VRS-SO, SORAG, Static-99, MnSOST-R, and RM2000/S) and global psychopathy (PCL/PCL-R, PPI/PPI-R, SRP, LSRP and others) (r = 0.33; 95% CI 0.21 - 0.44; p < 0.001). Heterogeneity was high (I² = 92.28%).	The analyses revealed that a greater emphasis on meanness in psychopathy scales significantly strengthened the relationship (β = 0.0050; p < 0.05), as did disinhibition (β = 0.0032; p < 0.01). However, boldness acted as a negative moderator (β = −0.0030; p < 0.001), reducing the strength of the link between psychopathy and sexually aggressive behaviors.
Mokros et al. (2010) [13]	Sadistic sexual offenders scored (DSM-IV-TR/ICD-10 and SSSS) significantly higher on psychopathy (PCL-R) than non-sadistic offenders (21.49 vs. 17.12; t = 2.95; p = 0.004; d = 0.59). Total psychopathy (PCL-R) exhibited a moderate positive correlation with sexual sadism (SSS) (r = 0.29; p = 0.002; 95% CI 0.09 - 0.46).	The interpersonal facet of the PCL-R (Factor 1), including superficial charm, grandiosity, and manipulation, showed a non-significant association with sexual sadism (r = 0.14; p = 0.085). In contrast, the affective facet (Factor 2), involving lack of remorse and empathy deficits, showed a significant positive correlation (r = 0.30, p = 0.002; 95% CI: 0.10 - 0.47) and was the strongest predictor in the MIMIC model (β = 0.27). The lifestyle/stimulation-seeking facet (Factor 3) showed no significant association (r = −0.07; p = 0.266). Finally, the antisocial/disinhibition facet (Factor 4) showed a significant but moderate correlation (r = 0.22; p = 0.019; 95% CI: 0.01 - 0.40), with a smaller contribution in the structural model (β = 0.16).
O’Connell et al. (2019) [14]	The meta-analysis found a significant positive correlation between total psychopathy (PCL-R) and sadism (r = 0.24; 95% CI 0.16 - 0.34; p < 0.001), although heterogeneity across studies was high (I² = 84.50%). There was a significant positive correlation between total psychopathy and sexual sadism, with an r-value of 0.22 (95% CI: 0.12 - 0.32) and a statistically significant magnitude (Z = 4.24; p < 0.001). By contrast, non-sexual sadism was more strongly associated with psychopathy (r = 0.36; 95% CI: 0.17 - 0.58; Z = 3.57; p = 0.001).	Factor 1 showed a small-to-moderate significant correlation (r = 0.25; 95% CI: 0.09 - 0.44; Z = 3.01; p < 0.01), with moderate heterogeneity (Q = 15.44; p = 0.03; I² = 54.70%) and stability in sensitivity analyses (r = 0.16 - 0.29). Similarly, Factor 2 showed an equivalent association (r = 0.26; 95% CI: 0.08 - 0.46; Z = 2.82; p < 0.01), also with moderate heterogeneity (Q = 18.52; p < 0.01; I² = 62.2%) and stability in leave-one-out analyses (r = 0.20 - 0.31).
Papagathonikou et al. (2025) [36]	Sexually violent offenders exhibited significantly higher psychopathy scores (PCL-R) than non-sexually violent offenders (27.28 ± 3.32 vs. 20.63 ± 5.59; p = 0.019), while sexual sadists also scored higher than non-sexual sadists (28.40 ± 2.23 vs. 20.70 ± 5.41; p = 0.005). Psychopathy was moderately correlated with sexual sadism (r = 0.551; p < 0.01) and weakly correlated with trait sadism (r = 0.281; p < 0.05). Furthermore, sexual sadism (SESAS) significantly predicted psychopathy (β = 0.516, R² = 0.334; p < 0.001), explaining 33.40% of the variance in PCL-R scores.	
Balcioglu et al. (2023) [37]	Pearson's correlation analysis revealed no significant correlation between total psychopathy (LSRP) and the degree of violence in sexual offenses (VPCO) (r = −0.13; p > 0.05). Similarly, multivariate ordinal regression revealed that total psychopathy was not an independent predictor of increased violence during sexual assault (β = 0.003; SE = 0.032; Wald = 0.007; p = 0.933; 95% CI: −0.060 to −0.065). The overall model was significant (χ² = 41.185; p < 0.001) and explained 50.5% of the variability in level of violence (Nagelkerke R² = 0.505); however, no significant multicollinearity issues were observed (VIF <5).	Among the psychopathy dimensions assessed with the LSRP, only secondary psychopathy was significantly associated with greater violence during sexual assault, showing a positive correlation with the violence index (r = 0.35; p < 0.05). Multivariate analysis confirmed secondary psychopathy as an independent predictor of violence (β = 0.061, 95% CI: 0.003 - 0.118; p = 0.038), whereas primary psychopathy was not significantly associated with violence in either correlation or regression analyses (p > 0.05).
Eher et al. (2015) Study 2 [38]	Psychopathy (PCL-R) showed a moderate positive correlation with sexual sadism (SeSaS) (r = 0.31; p ≤ 0.001), although weaker than the associations observed with established risk assessment tools (SORAG and Static-99; *r* = 0.53 - 0.79). The PCL-R demonstrated moderate predictive accuracy for violent recidivism (AUC = 0.72, 95% CI: 0.67 - 0.77; *p* < 0.001), outperforming SeSaS (AUC = 0.56) and clinical diagnosis of sadism (AUC = 0.48). In Cox regression analysis, PCL-R remained an independent predictor of violent recidivism (B = 1.07; 95% CI: 1.05 - 1.09; *p* < 0.001), whereas SeSaS and DSM-IV-TR sadism diagnosis did not provide additional predictive value.	
Brown et al. (1997) [39]	The distribution of rapist subtypes differed significantly between psychopaths and non-psychopaths (χ² = 12.73; p < 0.01). However, only one of 21 psychopaths (4.80%) was classified as a sexual-sadistic rapist compared to six of 39 non-psychopaths (15.40%). Furthermore, six of seven sadistic rapists (85.70%) were not classified as psychopaths. Consistently, sadistic sexual offenders did not exhibit the highest psychopathy scores. They had a mean score of 24.6 ± 7 on the PCL-R, which was lower than that of opportunistic rapists (28.1 ± 4.6) and similar to that of predominantly angry rapists (25.60 ± 6.70). The mean scores were 9.0 ± 3.60 for Factor 1 and 12 ± 3.70 for Factor 2 of the PCL-R (PCL-R; MTC:R3).	No specific association was identified between facets of psychopathy (PCL-R) and sexual sadism (MTC:R3). The total PCL-R score differed significantly among rapist subtypes (F = 4.80; p < 0.002). However, these differences were due to opportunistic rapists scoring higher than non-sadistic and vindictive rapists (p < 0.05 and p < 0.01, respectively), with no significant differences for the sexual-sadistic subtype. Similarly, Factor 1 showed overall differences (F = 3.40; p < 0.02), but only opportunistic rapists scored higher than vindictive rapists (p < 0.01). Sexual sadistic rapists had a mean score of 9 ± 3.60. Overall differences were also observed for Factor 2 (F = 2.8; p < 0.03), though post hoc analyses did not identify significant differences among the subtypes, including the sexual sadistic subtype, which had a mean score of 12 ± 3.70 (PCL-R; MTC:R3). Taken together, these results suggest that the total PCL-R score and its factors are not specifically associated with sexual sadism.

Global Association Between Psychopathy and Sadism

Available meta-analyses confirm a positive, albeit moderate, association between psychopathy and sadism. O’Connell et al. [14] reported a significant correlation between total psychopathy (PCL-R) and general sadism (r = 0.24). This association was weaker for sexual sadism (r = 0.22) and stronger for nonsexual sadism (r = 0.36). Similarly, Packer et al. [43] found a moderate association between sexual sadism and overall psychopathy (r = 0.33). However, there was very high heterogeneity across studies (I² = 92.28%), suggesting that this relationship varies substantially depending on context and instruments used. At the level of individual studies, Mokros et al. [13] and Robertson et al. [33] reported positive, significant correlations (r = 0.29 and r = 0.16-0.35, respectively), while Lobbestael et al. [42] and Lassche et al. [40] found moderate-magnitude associations (r = 0.25 and r = 0.53, respectively).

However, these findings are not universal. Balcioglu et al. [37] did not observe a significant correlation between total psychopathy and the degree of violence in sexual offenses (r = −0.13). Reidy et al. [41] concluded that psychopathic traits explained only 3% of the variance in sadism (R² = 0.03).

Differences Between Groups: Sadistic vs. Non-sadistic Subjects

Mokros et al. [13], Darjee [34], Holt et al. [35], and Papagathonikou et al. [36] found that individuals with sadism (whether sexual or personality-based) scored significantly higher on the PCL-R than non-sadistic individuals. Woodworth et al. [31] reinforced this idea by showing that 67% of sadistic subjects exhibited high levels of psychopathy and that 58% of subjects with high psychopathy scores exhibited sexual sadism.

However, Brown et al. [39] reported an opposite pattern: only 4.8% of psychopaths were classified as sadistic sexual offenders compared to 15.4% of non-psychopaths. Additionally, sadistic offenders did not exhibit the highest psychopathy scores compared to other offender subtypes. This study is one of the clearest exceptions within the reviewed literature.

The Role of the Factors and Facets of Psychopathy (PCL-R)

Findings regarding which specific psychopathic traits are associated with sadism are mixed. Several studies have highlighted the role of factor 2 (antisocial/impulsive lifestyle) and its facets. For example, Mokros et al. [13] found that the affective facet (factor 2) was the strongest predictor of sexual sadism (β = 0.27). However, Cardona et al. [30] identified the antisocial facet (factor 4) as the most robust predictor of both sexual and nonsexual aggression.

Conversely, other studies attribute greater weight to factor 1 (interpersonal-affective). Darjee [34] found that factor 1 correlated more strongly with the dimensional measure of sexual sadism, whereas factor 2 correlated more strongly with the DSM-IV categorical diagnosis. In study 1, Robertson et al. [33] observed significant associations with facets 1 (interpersonal), 3 (impulsivity), and 4 (antisocial), but not with facet 2 (affective). In study 2 (PCL-R and MTC:R3), however, they found associations with facets 1, 2, and 4, but not with facet 3.

Finally, Brown et al. [39] found no specific association between PCL-R facets and the sexual sadistic subtype. Holt et al. [35] reported similar correlations between both factors and sadism (r = 0.45 and r = 0.46) without clear differentiation.

Psychopathy, Sadism, and Associated Violence/Aggression

Rosenberg et al. [29] found that child sexual offenders who used physical violence during their assaults had significantly higher PCL-R scores than those who did not. Both factors were associated with violence. However, Balcioglu et al. [37] challenged this conclusion. Among the psychopathy dimensions (LSRP), only secondary psychopathy was significantly associated with greater violence during sexual assault (r = 0.35, β = 0.061, p = 0.038). Primary psychopathy showed no significant relationship.

Everyday Sadism and Dimensional Models (Dark Tetrad)

Studies of the general population beyond the forensic context show very high latent correlations between subclinical psychopathy and everyday sadism. Blötner et al. [18] reported extremely high latent correlations between psychopathy and sadism measures (ρ = 0.94-0.98), suggesting a possible conceptual overlap between the two constructs. Within the TriPM model, the "Meanness" dimension was most strongly associated with sadism, followed by "Disinhibition," while "Boldness" showed weak associations. This pattern aligns with Packer et al.'s [43] findings that "meanness" and "disinhibition" strengthen the association between psychopathy and sexual sadism, while "boldness" acts as a negative moderator, weakening that relationship.

Specific Dimensions: Impulsivity, Emotional Coldness, and Dominance

Lobbestael et al. [42] provided an important nuance by differentiating between types of sadism. Self-reported sadism was primarily associated with self-centered impulsivity (SCI), while affective coldness was the most significant predictor of behavioral sadism (e.g., number of insects crushed). Affective coldness was also associated with greater positive affect following aggression and lower guilt. On the other hand, fearless dominance predicted only lower guilt and was not related to sadism itself.

Sadistic Personality Disorder (SPD) and Its Relationship With Antisocial Personality Disorder

Berger et al. [32] examined the relationship between sadistic personality disorder (SPD) and antisocial personality disorder (ASPD). Using the Diagnostic and Statistical Manual of Mental Disorders, Third Edition, Revised (DSM-III-R) criteria, 42.1% of patients with SPD met the criteria for ASPD. However, this difference was not statistically significant. Using the International Classification of Diseases, 10th Revision (ICD-10) criteria for dissocial disorder, the proportion increased to 57.9%, which was statistically significant (p < 0.05). The authors concluded that SPD does not have its own distinct factor structure, but rather is a subdimension of the same antisocial construct, characterized by persistent cruelty and a dominant orientation.

Predictive Validity for Violent Recidivism

Regarding predictive utility, Eher et al. [38] demonstrated that although the PCL-R and the Sexual Sadism Scale (SeSaS) moderately correlated (r = 0.31), the PCL-R's predictive power for violent recidivism (area under the curve (AUC) = 0.72) was superior to that of both the SeSaS (AUC = 0.56) and the clinical diagnosis of sadism (AUC = 0.48), the latter of which was not significant. In Cox regression models, the PCL-R remained an independent predictor of violent recidivism; neither the DSM-IV-TR diagnosis of sadism nor the SeSaS provided additional predictive power.

Nosological Implications of Sadism: An Independent Dimension or a Manifestation of Psychopathy and Antisocial Personality Disorder?

The findings of this review support the idea that sadism is consistently linked to psychopathy. However, the strength of this link depends on the context (clinical, forensic, or community) and the instruments used. Meta-analyses [14,43] revealed significant positive correlations between the two constructs (r = 0.24, 95% CI: 0.16-0.34, p < 0.001; and r = 0.33, 95% CI: 0.21-0.44, p < 0.001). The association was stronger for nonsexual sadism (r = 0.36) than for sexual sadism (r = 0.22). Furthermore, individual studies showed that individuals with sadistic traits or a sadism diagnosis had higher psychopathy scores, particularly in forensic populations. However, the high heterogeneity among the studies (I² = 84.5%-92.3%) and the presence of conflicting results suggest that sadism cannot be considered an equivalent manifestation of psychopathy.

With regard to its nosological classification, the evidence suggests that sadism shares a common core with central dimensions of psychopathy, particularly cruelty, emotional insensitivity, disinhibition, and interpersonal dominance. The strongest associations were observed with dimensions related to lack of empathy and cruelty (ρ = 0.94-0.98 on latent measures of psychopathy and everyday sadism), while audacity showed weaker relationships or even negative moderating effects. These results support a dimensional conceptualization of sadism as a partially overlapping but potentially distinguishable construct within contemporary models such as HiTOP and the dimensional approaches of the DSM-5. Therefore, although sadism shares characteristics with antisocial and psychopathic disorders, current evidence does not support reducing it entirely to these diagnoses, but rather considers it an independent dimension related to cruelty and interpersonal aggression.

Risk of Bias

The methodological quality of the included observational studies was evaluated using an adapted version of the NOS [25]. The results are presented in Table 3, and scores ranged from 7 to 16 points, indicating predominantly moderate to high quality. The highest-quality studies scored 16 out of 16 points, while most scored between 11 and 14 points. The main strengths were the use of validated instruments to assess psychopathy and sadism, adequate measurement of outcomes, and appropriate statistical analyses. The most common limitations were the use of non-probability or convenience samples, limited information on non-responders, the absence of statistical power analyses, and limited comparability between groups. These aspects should be considered when interpreting the results.

**Table 3:** Quality assessment of included studies using the Newcastle-Ottawa Scale (NOS). PCL-R: Psychopathy Checklist-Revised; DSM-IV/DSM-IV-TR/DSM-5: Diagnostic and Statistical Manual of Mental Disorders; SeSaS: Sexual Sadism Scale; SSSS: Severe Sexual Sadism Scale; MIDSA: Multidimensional Inventory of Development, Sex, and Aggression; MTC:CM3/MTC:R3: Massachusetts Treatment Center Classification Systems (version 3); MCMI-II: Millon Clinical Multiaxial Inventory-II; PDE: Personality Disorder Examination; IPDE: International Personality Disorder Examination; SORAG: Sex Offender Risk Appraisal Guide; Static-99: actuarial risk assessment tool for sexual recidivism; CFA: confirmatory factor analysis; EFA: exploratory factor analysis; MIMIC: multiple indicators multiple causes; ANCOVA: analysis of covariance; ANOVA: analysis of variance; AUC: area under the curve; ICC: intraclass correlation coefficient. Scores were classified as high quality (13-16), moderate quality (9-12), and low quality (≤8).

Study	Clearness of stated aim (0-2)	Sample selection				Comparability		Outcome		NOS total score (Quality assessment) (0-16)
		Sample representativeness (0-2)	Sample size (0-2)	Non-respondents (0-2)	Exposure assessment (0-2)	Control of confounding factors (0-1)	Comparability of participants (0-1)	Assessment of the outcome (0-2)	Statistical tests (0-2)	NOS total (0-16)
Woodworth et al. (2013) [31]	1/2 - Extensive introduction (~27 references), but fewer than 10 directly addressing the specific research question.	1/2 - Sample drawn from the highest-risk third of the ISPIN/HROIP database; targeted non-probability sampling but appropriate for the study objective.	1/2 - n=139; exceeded the ≥100 threshold, although no a priori power analysis was reported.	0/2 - Substantial missing data (PCL-R available for only 70/139 participants); no response rate or comparison of missing cases reported.	2/2 - Official records, DSM-IV-TR diagnoses, and PCL-R used as reference standards.	1/1 - Bonferroni correction and post hoc analyses were applied to control Type I error.	0/1 - Exclusively male sample; no assessment of demographic balance between groups.	2/2 - Diagnoses established by clinicians using DSM-IV-TR criteria and PCL-R assessments.	2/2 - χ² tests, ANOVA, Goodman tests, Scheffé, Dunnett’s C, Tukey post hoc tests, η², and Cramer’s V were appropriately reported.	10/16
Darjee (2019) [34]	1/2 - Broad introduction, but no evidence of ≥10 references directly focused on the specific knowledge gap.	0/2 - Forensic clinical case series based on referred offenders; convenience sample.	0/2 - n=51; below the ≥100 threshold and no power calculation reported.	0/2 - No information provided on non-response rates or comparison of included versus excluded cases.	2/2 - SeSaS, PCL-R, and DSM-IV diagnoses were administered through structured clinical evaluations.	1/1 - Logistic regression incorporating multiple predictors and interaction terms.	0/1 - No assessment of demographic balance between comparison groups.	2/2 - Variables derived from structured clinical interviews and official records.	2/2 - Shapiro-Wilk tests, Mann-Whitney U tests, t-tests, Kendall’s τ, and logistic regression with effect sizes reported.	8/16
Cardona et al. (2018) [30]	1/2 - Strong introduction, although fewer than 10 references were directly focused on the primary research question.	0/2 - Convenience sample selected based on availability of records.	1/2 - N=302; adequate sample size but no formal power analysis reported.	0/2 - 227 files were excluded without analysis of comparability.	2/2 - PCL-R and DSM-5 criteria administered by trained evaluators with high reliability.	1/1 - Hierarchical regression analyses controlled for relevant variables.	0/1 - No evidence of demographic equivalence between groups.	2/2 - Standardized sexual aggression measures with adequate reliability.	2/2 - Hierarchical regressions, factor analyses, and correlations were appropriately reported.	9/16
Rosenberg et al. (2005) [29]	1/2 - Extensive theoretical background, but fewer than 10 references specifically addressed the primary hypothesis.	0/2 - Quota sampling based on institutional records.	1/2 - N=111; no statistical power calculation reported.	0/2 - No information on excluded cases or comparability provided.	2/2 - PCL-R administered by trained evaluators with high inter-rater reliability.	0/1 - No adjustment for potential confounding variables.	0/1 - No assessment of balance across the three comparison groups.	2/2 - Variables obtained from official records under explicit criteria.	1/2 - ANOVA and correlation analyses were appropriate, but effect sizes and multiple-comparison corrections were not reported.	7/16
Papagathonikou et al. (2025) [36]	1/2 - Adequate references, but fewer than 10 were directly focused on the specific research question.	1/2 - Clinical sample recruited from NHS hospitals and a prison setting; convenience-based but relevant to the objective.	1/2 - n=59; no a priori power analysis was conducted.	0/2 - Three participant withdrawals were reported without a comparative analysis of completers versus non-completers.	2/2 - PCL-R, SESAS, and ASP were validated and adequately described.	1/1 - Multiple regression models simultaneously controlled for predictors.	0/1 - No documented balance between groups.	2/2 - Clinical interviews and official records reviewed by professionals.	2/2 - Correlations, regressions, Student’s t-tests, and thematic analyses were appropriately described.	10/16
Lobbestael et al. (2020) [42]	2/2 - More than 10 references directly relevant to psychopathy and sadism.	1/2 - Community sample of males recruited through advertisements; non-probability sampling.	2/2 - An a priori power analysis was conducted, and the sample size was deemed adequate.	1/2 - Exclusion of nine participants was reported, but no formal comparison with the excluded individuals.	2/2 - Validated instruments with adequate psychometric properties.	1/1 - Baseline values were controlled through regression analyses and ANCOVA.	0/1 - No sex balance across groups.	2/2 - Standardized behavioral and psychometric measures.	2/2 - Regression analyses, ANOVA, and moderation analyses with effect sizes reported.	13/16
Lassche et al. (2024) [40]	2/2 - More than 10 references directly linked to the study topic.	1/2 - University convenience sample.	2/2 - Sample size justified through G*Power analysis.	1/2 - Two exclusions were reported without a formal post-exclusion comparison.	2/2 - Validated questionnaires and EEG as an objective measure.	1/1 - Hierarchical regression and sequential moderation analyses were performed.	0/1 - Marked imbalance between male and female participants.	2/2 - EEG and validated questionnaires administered under standardized procedures.	2/2 - Correlations and regressions reported with 95% confidence intervals and coefficients.	13/16
Berger et al. (1999) [32]	2/2 - More than 10 relevant references on SPD, sadism, and antisocial personality.	1/2 - Consecutive institutionalized sample; non-probabilistic but relevant.	0/2 - N=70 with no power analysis.	1/2 - Only one refusal was reported; no comparison between the participants and non-participants.	2/2 - IPDE interviews, clinical assessments, and official records used.	0/1 - No multivariable control of confounding factors.	0/1 - No assessment of comparability between SPD and non-SPD groups.	2/2 - Diagnoses based on clinical consensus and structured interviews.	2/2 - χ² analyses, factor analyses, and diagnostic parameters appropriately reported.	10/16
Robertson et al. (2014) [33] - Study 1	2/2 - Clear objective and more than 10 relevant references.	1/2 - Non-probability clinical-forensic sample.	1/2 - Adequate sample size, although no power analysis was reported.	0/2 - No information regarding non-response provided.	2/2 - Validated MIDSA and PCL-R instruments.	1/1 - Multivariable regression analyses were conducted.	0/1 - No assessment of equivalence between groups.	2/2 - Objective archival assessments conducted by blinded evaluators.	2/2 - Correlations, factor analyses, and regression analyses were appropriately reported.	11/16
Robertson et al. (2014) [33] - Study 2	2/2 - Clear objective and more than 10 relevant references.	1/2 - Non-probability clinical-forensic sample.	1/2 - Adequate sample size, although no power analysis was reported.	0/2 - No information regarding non-response provided.	2/2 - Validated MTC:CM3, MTC:R3, and PCL-R instruments.	1/1 - Multivariable regression analyses were conducted.	0/1 - No assessment of equivalence between groups.	2/2 - Archival coding procedures demonstrated adequate reliability.	2/2 - CFA, correlations, and regression analyses appropriately reported.	11/16
Brown et al. (1997) [39]	2/2 - Clear objectives and strong theoretical foundation.	1/2 - Prison sample with overrepresentation of serious offenders.	1/2 - N=60 without a formal power calculation.	1/2 - Partial reporting of participation and attrition.	2/2 - PCL-R and other standardized measures were used.	1/1 - Statistical control for potential confounding variables.	1/1 - Groups originated from the same population and were assessed under identical criteria.	2/2 - Interviews and records demonstrated adequate reliability.	2/2 - ANOVA, χ² tests, correlations, and post hoc analyses were appropriately conducted.	13/16
Blötner et al. (2025) [18]	2/2 - Clearly defined objectives and hypotheses supported by the literature.	1/2 - Predominantly female student samples, limiting representativeness.	2/2 - Two independent samples with sufficient size for complex CFA models.	1/2 - Non-response rates were not described in detail.	2/2 - Multiple validated psychopathy and sadism instruments.	1/1 - Preregistration and correction for multiple comparisons.	1/1 - Internal replication using the same methodology.	2/2 - Broad set of validated psychological and behavioral measures.	2/2 - CFA, ICCDE, correlations, and robust network analyses.	14/16
Mokros et al. (2010) [13]	2/2 - Clear objectives and strong theoretical rationale.	1/2 - Forensic patients from a single hospital; limited representativeness.	1/2 - N=100 without a formal power analysis.	1/2 - Missing data reported without comparison to non-participants.	2/2 - PCL-R and SSSS demonstrated adequate reliability.	1/1 - Multivariable models controlled latent dimensions.	1/1 - Homogeneous procedures were applied to all participants.	2/2 - Structured measures with adequate validity and reliability.	2/2 - EFA, CFA, MIMIC models, and fit indices were appropriately reported.	13/16
Holt et al. (1999) [35]	2/2 - Clear hypotheses and comprehensive literature review.	1/2 - Random prison records from a maximum-security institution, although the final sample was restricted.	1/2 - No a priori sample size calculation; power assessed post hoc.	1/2 - Attrition reported without formal comparison.	2/2 - Validated PCL-R, MCMI-II, and PDE instruments.	1/1 - Statistical adjustment for age, race, and education.	1/1 - No significant demographic differences between groups.	2/2 - Independent blinded evaluators with high inter-rater agreement.	2/2 - ANOVA, χ² tests, correlations, and power analyses were appropriately conducted.	13/16
Eher et al. (2015) [38] - Study 2	2/2 - Clearly defined and well-supported objective.	2/2 - Large consecutive sample representative of the target population.	2/2 - N=768, sufficient for the analyses performed.	2/2 - Rejection rate <2% clearly reported.	2/2 - SeSaS, PCL-R, SORAG, and Static-99 demonstrated high reliability.	1/1 - Cox regression models controlled for multiple relevant variables.	1/1 - Stratification by offender type maintained comparability.	2/2 - Official records assessed by blinded evaluators.	2/2 - AUC, DeLong tests, Cox regression, and survival analyses appropriately applied.	16/16
Balcioglu et al. (2023) [37]	2/2 - Clear and well-supported objectives.	1/2 - Specific forensic sample not representative of all sexual offenders.	1/2 - No sample size calculation reported.	0/2 - No information regarding non-response provided.	2/2 - Validated instruments with adequate internal consistency.	1/1 - Ordinal regression adjusted for relevant variables.	1/1 - Sociodemographic and offense-related variables were assessed and controlled.	2/2 - Violence ratings conducted by blinded assessors (ICC = 0.88).	2/2 - Correlations and ordinal regression analyses were appropriately performed.	12/16

AMSTAR-2

The methodological quality of the two included meta-analyses [14,43] was assessed using the AMSTAR-2 tool [27], and the results are presented in Table 4. Both studies were classified as having low methodological quality due to the absence of duplicate study screening, failure to assess and account for risk of bias when interpreting results, and omission of funding sources for included studies. However, both studies had significant strengths, including a clearly defined PICO (population, intervention, comparison, outcome) question, a comprehensive literature search, the use of appropriate meta-analytic methods, an assessment of publication bias, and disclosure of conflicts of interest. Additionally, one meta-analysis had a registered protocol and performed duplicate data extraction, and the other provided a list of excluded studies and the rationale for their exclusion.

**Table 4:** Methodological quality assessment of included studies according to the AMSTAR-2 criteria. AMSTAR-2: A Measurement Tool to Assess Systematic Reviews 2; PICO: population, intervention, comparison, outcome.

AMSTAR 2 domain	O'Connell et al. [14]	Packer et al. [43]
PICO clearly defined	Yes	Yes
Protocol registered a priori	Yes	No
Justification of study designs	Yes	Yes
Comprehensive literature search	Yes	Yes
Study selection in duplicate	No	No
Data extraction in duplicate	Yes	No
List of excluded studies with justification	No	Yes
Adequate description of included studies	Yes	Yes
Risk of bias assessment	No	No
Funding sources of the included studies reported	No	No
Appropriate meta-analytic methods	Yes	Yes
Impact of risk of bias assessed	No	No
Risk of bias considered in interpretation	No	No
Heterogeneity adequately explained	Yes	Yes
Publication bias assessed	Yes	Yes
Conflicts of interest reported	Yes	Yes
Overall AMSTAR 2 rating	Low	Low

RoB 2

The risk of bias in the experimental study was assessed using the RoB 2 tool [26], and the results are presented in Table 5. The study was rated as having an overall risk of "some concerns," primarily due to limited information on the randomization process and potential biases related to selective reporting of outcomes. Conversely, the domains related to deviations from the intervention, incomplete data, and outcome measurement were rated as having a low risk of bias, indicating adequate internal validity in these areas.

**Table 5:** Risk of bias (RoB 2) assessment of included studies.

Study	Randomization	Deviations from the intervention	Incomplete data	Outcome measurement	Selective reporting	Overall risk	Methodological justification
Reidy et al. (2011) [41]	Some concerns	Low	Low	Low	Some concerns	Some concerns	Laboratory experimental study with limited information on allocation concealment

GRADE Evidence Profile

We assessed the evidence profile using the GRADE methodology [28], and the results are presented in Table 6. Due to risk of bias, high heterogeneity, imprecision, and possible publication bias, the certainty of the evidence was very low for most outcomes. However, meta-analyses revealed a positive, significant correlation between psychopathy and sadism (r = 0.24-0.33; p < 0.001). Evidence on subclinical everyday sadism was of low quality. The relationship between psychopathy and violence during sexual assault was inconsistent, preventing estimation of a combined effect.

**Table 6:** GRADE assessment of the quality of evidence on the association between psychopathy and sadism. GRADE: Grading of Recommendations Assessment, Development, and Evaluation; I²: statistical heterogeneity index; r: correlation coefficient; d: Cohen’s effect size; ⨁⨁⨁⨁: high; ⨁⨁⨁◯: moderate; ⨁⨁◯◯: low; ⨁◯◯◯: very low; ASP: Assessment of Sadistic Personality; CAST: Comprehensive Assessment of Sadistic Tendencies; CI: confidence interval; DSM: Diagnostic and Statistical Manual of Mental Disorders; LSRP: Levenson Self-Report Psychopathy Scale; MIDSA: Multidimensional Inventory of Development, Sex, and Aggression; MCMI-II: Millon Clinical Multiaxial Inventory-II; NOS: Newcastle-Ottawa Scale; NS: not statistically significant; PCL-R: Psychopathy Checklist-Revised; SeSaS: Severe Sexual Sadism Scale; SRP-4: Self-Report Psychopathy Scale-4; SSIS: Short Sadistic Impulse Scale; TriPM: Triarchic Psychopathy Measure; VAST: Varieties of Sadistic Tendencies. Footnotes: ¹ Downgraded for risk of bias due to methodological limitations. ² Downgraded for a very serious inconsistency because of substantial heterogeneity and conflicting findings. ³ Downgraded for likely publication bias due to predominance of positive findings. ⁴ Downgraded for imprecision because of small sample sizes and wide confidence intervals. ⁵ Downgraded for inconsistency due to variability in effect sizes. ⁶ No downgrade for risk of bias because of high methodological quality. ⁷ Downgraded for inconsistency due to wide variability in correlations. ⁸ Downgraded for a very serious inconsistency because of directly contradictory findings.

Outcome	No. of studies	Study design	Risk of bias (design limitations)	Inconsistency	Indirectness	Imprecision	Publication bias	Estimated relative effect (95% CI)	Quality of evidence (GRADE)	Importance
Overall association between psychopathy and sadism - Total PCL-R vs. global sadism scales	2 meta-analyses (34 studies)	Meta-analysis of observational studies	⬇ −1 serious¹. Convenience samples; absence of independent dual extraction in meta-analyses; inadequate control of confounders; AMSTAR-2 rating LOW	⬇⬇ −2 very serious¹. I² = 84.5-92.3%; unexplained clinical, methodological, and construct heterogeneity	No serious limitation	No serious limitation	⬇ −1 likely³. Predominance of positive findings; negative/null studies minority; no formal funnel plot analysis	O’Connell et al. [14]: r = 0.24; 95% CI: 0.16 to 0.34; p < 0.001. Packer et al. [43]: r = 0.33; 95% CI: 0.21 to 0.44; p < 0.001	⊕◯◯◯VERY LOW	Critical
Sexual sadism and psychopathy - DSM / SeSaS / MIDSA vs. PCL-R in forensic samples	12 studies	Observational cross-sectional/retrospective cohort	⬇ −1 serious¹. Convenience samples; lack of a priori power calculations; limited comparability between groups; mean NOS ≈ 11/16	⬇⬇ −2 very serious². I² = 84.5-92.3% in subgroup analyses; conflicting findings, including opposite direction in Brown (1997)	No serious limitation	⬇ −1 serious⁴. Several studies with small samples (n = 41-70), broad confidence intervals, and a possible absence of effect	⬇ −1 likely³. Most studies reported positive associations; limited negative/null findings	Individual studies: r = 0.16 to 0.35. Meta-analysis subtotal: r = 0.22; 95% CI: 0.12 to 0.32. Exception: Brown et al. [39] reported the opposite direction	⊕◯◯◯VERY LOW	Critical
Non-sexual sadism and psychopathy - SSIS / MCMI-II / ASP vs. PCL-R or self-report measures	4 studies	Observational cross-sectional/experimental	⬇ −1 serious¹. Convenience samples; absence of power analysis in some studies	⬇ −1 serious⁵. Variability in effect sizes, although more consistent direction compared with sexual sadism	No serious limitation	No serious limitation	⬇ −1 likely³. Predominance of statistically significant positive studies	r = 0.36; 95% CI: 0.17-0.58; p = 0.001. Stronger association than the sexual sadism subgroup	⊕◯◯◯VERY LOW	Important
Subclinical everyday sadism and psychopathy - VAST / CAST / ASP / SSIS vs. LSRP, SRP-4, TriPM in community samples	3 studies	Observational cross-sectional/experimental	No downgrade⁶. Blötner 2025 and Lassche 2024: preregistration, a priori power analysis, internal replication; NOS 13-14/16	⬇ −1 serious⁷. Wide variability in correlations (r = 0.03-0.98); possible conceptual overlap between psychopathy and everyday sadism measures	No serious limitation	No serious limitation	No relevant concern identified	Blötner et al. [18]: ρ latent = 0.94 to 0.98. Lassche et al. [40]: r = 0.53. Reidy et al. [41]: R² = 0.03; p = NS	⊕⊕◯◯LOW	Important
Violence during sexual assault and psychopathy - PCL-R / LSRP vs. violence index during assault	3 studies	Observational cross-sectional/retrospective cohort	⬇ −1 serious¹. Convenience samples; lack of adjustment for confounders; heterogeneous methodological quality	⬇⬇ −2 very serious⁸. Directly contradictory findings: Rosenberg (higher PCL-R among violent offenders) vs. Balcioglu (negative non-significant association)	No serious limitation	⬇ −1 serious⁴. Small samples; wide confidence intervals; opposite directions between studies	⬇ −1 likely³. No formal asymmetry testing; predominance of positive findings	Rosenberg et al. [29]: Higher PCL-R scores in violent offenders (p < 0.0001). Balcioglu et al. [37]: r = − 0.13, p > 0.05. Pooled effect not estimable due to inconsistency	⊕◯◯◯VERY LOW	Critical

Discussion

This systematic review revealed a positive and consistent association between psychopathy and sadism, as supported by observational studies and meta-analyses. Generally, individuals with higher levels of psychopathy exhibit higher levels of sadism, especially in forensic settings and among sex offenders. However, the strength of this relationship varied across studies due to methodological differences, the populations assessed, and the measurement instruments used, resulting in conflicting results. Furthermore, the evidence indicated that certain dimensions of psychopathy, particularly antisocial, affective, and disinhibition traits, showed more consistent associations with sadistic behaviors than other facets. Associations were also observed between subclinical psychopathy and everyday sadism in non-forensic populations, though there was considerable conceptual heterogeneity. Overall, the quality of the evidence was predominantly low or very low according to GRADE due to high risk of heterogeneity, predominance of observational designs, and various methodological limitations. Therefore, the findings should be interpreted with caution.

The two meta-analyses included in this review [14,43] (19 samples; N = 5,161 and 15 studies; N = 3,074) were the most robust quantitative anchors. Both reported medium effect sizes (r = 0,24 - 0,33), consistent with the correlation coefficients obtained in the included individual studies. These values were comparable to those reported for psychopathy and proactive aggression. This positions sadism as a correlate of psychopathy, comparable in significance to other classic behavioral indicators of the construct.

The Differential Contribution of Psychopathy Facets

Studies agree that the affective and interpersonal facets of psychopathy are the best predictors of sadism, especially sexual and everyday sadism. Traits such as emotional insensitivity, lack of remorse, and grandiosity are more strongly associated with sadism than other psychopathic traits [13,33]. This finding supports Blair’s model, which posits that a low emotional response to others' fear facilitates aggression and the learning of harm as a source of pleasure. Similarly, the affective facet independently predicts various types of sadism, even when controlling for the total PCL-R score. This suggests that evaluating only the overall score may underestimate the risk of sadism when traits such as impulsivity or antisocial behavior predominate [30]. Finally, primary psychopathy, linked to the affective-interpersonal core, is more strongly associated with violence than secondary psychopathy [37], which reinforces the distinction between a cold, calculating, sadistic, psychopathic profile and a more impulsive, reactive one [32,35].

Is Sexual Sadism a Continuum of Everyday Sadism, or Are They Distinct Constructs?

The distinction between the two represents one of the most active conceptual debates in the literature [9,44]. Reviewed studies show that both are related to psychopathy, albeit in different ways. Sexual sadism, as observed in sex offenders, is primarily associated with the emotional and interpersonal facets of psychopathy [31,34,38]. It is linked to emotional coldness and a lack of empathy. In contrast, everyday sadism, as observed in community samples, maintained broader associations with the full spectrum of psychopathy, including its impulsive components [18,42].

Disinhibition was particularly related to instrumental sadism, while boldness showed weak links to sexual sadism [18]. Furthermore, electroencephalography (EEG) revealed a reduced empathic response to others’ pain in individuals with high levels of everyday sadism, suggesting a shared neurophysiological basis [40].

While some data support the idea of a continuum between psychopathy and sadism, other studies have shown that sadism predicts instrumental aggression even when controlling for psychopathy. These findings support the notion that sadism possesses independent explanatory value and cannot be solely attributed to psychopathy.

Future Research Directions

The findings of this review identify at least three priorities for future research. First, longitudinal studies are needed to examine the co-development of psychopathy and sadism from childhood through adolescence, as the existing etiological models are primarily cross-sectional. Second, research on female participants must be expanded because evidence regarding the expression of sadism in women with psychopathic traits is virtually nonexistent. Third, future primary studies should incorporate multivariable statistical controls and matched-sampling designs to account for potential confounding factors, including histories of violence, antisocial behavior, personality disorders, and other psychopathological conditions. Such approaches would help clarify the independent contribution of sadism beyond overlapping psychopathic and antisocial traits.

Limitations

One of the main limitations of the study is the high methodological heterogeneity among the included studies (I² = 84-92%), which is due to differences in study designs, psychometric instruments, definitions of sadism, and types of samples analyzed. Many studies used small, predominantly forensic or prison-based samples, which limits the generalizability of the results to the general population. Additionally, several studies had inadequate control of relevant confounding variables, such as a history of violence, other personality disorders, or environmental factors. Most studies were observational and cross-sectional and therefore could not establish causal relationships between psychopathy and sadism. Some experimental studies were also found to have a moderate risk of bias due to a lack of clear randomization and pre-registered protocols.

## Conclusions

This systematic review revealed a consistent association between psychopathy and sadism, particularly sexual sadism and sadistic behaviors, in forensic populations. The available evidence indicated that the magnitude of this relationship was small to moderate, though there was considerable heterogeneity among the studies. Furthermore, the results suggested that the strongest associations with sadism were found in dimensions related to disinhibition, antisocial traits, cruelty, and empathic deficits, while findings regarding affective-interpersonal facets were inconsistent. However, the overall quality of the evidence was predominantly low or very low due to methodological limitations, small sample sizes in several studies, observational designs, and high clinical and psychometric heterogeneity. Consequently, while the findings support the existence of a relationship between psychopathy and sadism, this relationship should be interpreted with caution.
